# Tape Worm

**Published:** 1874-11

**Authors:** 


New Treatment for Tape-Worm.—* * * I made no preliminary provisions, further than to forbid the patient from taking any breakfast the day I intended removing the worm, and giving him a large dose of Rochelle salts the preceding night. At 10 o'clock in the morning he had the following at one dose:

| | |
|---|---|
| Take, Bark of Pomegranate Root, . . | ½ ounce. |
| Pumpkin seed, . . . . . | ½ drachm. |
| Ethereal Extract of Male Fern, . | 1 drachm. |
| Powdered Ergot, . . . . | ½ drachm. |
| Powdered Gum Arabic, . . . | 2 drachms. |
| Croton Oil, . . . . . . . | 2 drops. |

The pomegranate bark and pumpkin seed were thoroughly bruised, and, with the ergot, boiled in eight ounces of water for fiteen minutes, then strained through a coarse cloth. The croton oil was first well rubbed up with the acacia and extract male fern, and then formed into an emulsion with the decoction. In each case the worm was expelled, alive and entire, within two hours. No unpleasant effects followed.

One curious fact that I have noticed is that in each case the worm was passed with the head firmly fastened to the side of its body at about the widest part, from which it was with difficulty removed; also that the worm was twisted and doubled into various knots. In one specimen I have, although only fourteen feet long, counted and untied no less than forty-seven of such knots. I have no doubt that, to escape the effects of the medicine, in his distress, he fastens himself to his own body, and in this way losing his hold of the intestines, is driven forward with the other contents of the bowels.—*The Archives.*

